# Machine learning-based risk factor analysis and prediction model construction for mortality in chronic heart failure

**DOI:** 10.7189/jogh.15.04242

**Published:** 2025-09-12

**Authors:** Qian Xu, Ruicong Yu, Xue Cai, Guanjie Chen, Yueyue Zheng, Cuirong Xu, Jing Sun

**Affiliations:** 1 Zhongda Hospital, Southeast University, Nanjing, China; 2 School of Medicine, Southeast University, Nanjing, China; 3 Department of Respiratory and Critical Care, Zhongda Hospital, Southeast University, Nanjing, China; 4 Department of Critical Care Medicine, Zhongda Hospital, Southeast University, Nanjing, China; 5 Department of Geriatrics, Zhongda Hospital, Southeast University, Nanjing, China; 6 Department of Nursing, Zhongda Hospital, Southeast University, Nanjing, China; 7 Rural Health Research Institute, Charles Sturt University, Bathurst, Australia; 8 Data Science Institute, University of Technology Sydney, Sydney, Australia; 9 Heart Research Institute, University of Sydney, Sydney, Australia

## Abstract

**Background:**

Given the high global mortality burden of chronic heart failure (CHF) and the limitations of traditional risk prediction tools in accuracy and comprehensiveness, along with the potential of machine learning (ML) to improve prediction performance and the ability of a health ecology framework to systematically identify multi-dimensional risk factors, we aimed to develop an ML-based mortality risk prediction model for CHF and analyse its risk factors using a health ecology framework.

**Methods:**

We enrolled 489 CHF patients from the Jackson Heart Database, with all-cause mortality during a 10-year follow-up period designated as the outcome measure. Guided by a five-layer health ecology framework (individual traits, behavioural characteristics, interpersonal relationships, work/living conditions, and macro policies), we selected 58 variables for analysis. The cohort was split into 7:3 training/validation sets. Random forest (RF) and k-nearest neighbour (KNN) models identified mortality predictors after five oversampling techniques addressed data imbalance before modelling. We trained seven ML algorithms, validated them via 10-fold cross-validation, and compared them using accuracy, the area under the curve (AUC), and other metrics.

**Results:**

We identified 24 key factors: 19 for individual traits (age, body mass index (BMI), antihypertensive medication, hypoglycaemic medication, antiarrhythmic medication, systolic blood pressure, glycated haemoglobin, glomerular filtration rate, left ventricular ejection fraction, left ventricular diastolic diameter, left ventricular mass, high-density lipoproteins, low-density lipoproteins, triglycerides, total cholesterol, cardiovascular surgical history, mitral annular early diastolic peak velocity of motion); three for individual behavioural characteristics (dark greens intake, egg intake, and night-time sleep duration); and two for living and working conditions (favourite food shop at three-kilometre radius, proportion of poor people in the place of residence). The model constructed using synthetic minority over-sampling technique combined with edited nearest neighbours (SMOTE-ENN) processing and applying extreme gradient boosting (XGBoost) model was optimal, with an accuracy of 81.58%, an AUC value of 0.83, a precision of 0.87, a recall of 0.84, and an F1 value of 0.86 for the prediction of mortality at 10-year follow up.

**Conclusions:**

We systematically categorised CHF mortality risk factors by integrating health ecology theory and ML. The SMOTE-ENN and XGBoost model demonstrated high accuracy, though further optimisation is needed to enhance clinical utility in CHF risk prediction.

Chronic heart failure (CHF) encompasses a range of advanced cardiovascular disorders that arise from various aetiological factors and are characterised by a spectrum of clinical manifestations. They are marked by impaired contractile or diastolic function of cardiac myocytes, resulting in a relative or absolute reduction in cardiac output. The predominant clinical features of CHF include dyspnoea, varying degrees of physical activity limitation, and fluid retention, which can lead to pulmonary and circulatory congestion [1].

CHF significantly contributes to mortality among patients with advanced cardiovascular diseases [2]. The one-year mortality risk in the geriatric population ranges from 15% to 30%, while the five-year mortality risk can be as high as 75% and hospitalisation due to acute and recurrent heart failure is associated with an increased mortality risk [3–5]. Therefore, to reduce CHF-related deaths, it is essential to predict mortality risk from CHF and to determine associated risk factors to facilitate the development of targeted therapeutic interventions.

There is currently no reliable prediction scale or tool for assessing mortality risk in patients with CHF, with clinical assessments primarily depending on biological markers and health care professionals’ knowledge and experience. Existing clinical guidelines underscore the importance of early and accurate risk stratification for the prevention and management of cardiovascular diseases, particularly within the ‘health for all’ and ‘personalised medicine’ frameworks [6]. Therefore, developing a predictive model for mortality risk in CHF is of utmost significance. To do so, it is crucial to accurately and comprehensively identify risk factors and to utilise sophisticated, validated algorithms.

Prior research on CHF has predominantly focussed on isolated dimensions, such as biological markers [7,8], lifestyle choices [9,10], and health economics [11], rather than holistically examining the patients’ health-related and social circumstances. In this context, the health ecology model posits that health outcomes are influenced by the interplay between individuals and their environments [12]. It is structured around five levels: individual characteristics (*e.g.* age, gender, health status), individual behavioural traits (*e.g.* psychological, behavioural, and lifestyle factors), interpersonal relationships (*e.g.* family, community, and social networks), working and living conditions (*e.g.* socioeconomic status and access to health care services), and macro-level policies (*e.g.* local, national, and global policies) [12]. This framework situates individuals within a multidimensional, integrated environment, which is particularly relevant to the complexities associated with heart failure diseases.

Novel methodologies and technologies, such as artificial intelligence and data-driven electronic health record mining, can provide valuable insights into complex, large data sets and thus help inform precise, personalised medical decision-making and health management. Machine learning (ML) specifically has been applied extensively in the clinical management of cardiovascular diseases, with ML models often outperforming traditional models in predicting mortality risk and identifying associated factors across various medical conditions [13]. Research has shown that ML approaches can effectively predict the mortality rates of patients with hepatic encephalopathy and those with metastatic colorectal cancer [14,15], with evidence suggesting that ML can help with the diagnosis and risk assessment in cardiovascular diseases [16]. We therefore theorised that ML could also be useful in predicting CHF-related mortality. Leaning on the health ecology model, we sought to compare the capacity of various ML approaches to accurately and rapidly identify risk factors that may contribute to the mortality of CHF patients and develop tools to predict their ten-year mortality rate.

## METHODS

### Data source

We retrieved our data from the Jackson Heart Study database hosted by the National Heart, Lung, and Blood Institute, which comprises data on 3883 African American adults aged 35–84 years, with longitudinal data collected at baseline (V1) in 2000–04, first follow-up (V2) in 2005–08, and second follow-up (V3) in 2009–13 [17]. At baseline, 489 participants had CHF, and 3394 did not. Overall, 777 patients were lost during the first-year follow-up (V2) and an additional 462 during the second year (V3), leaving a final sample size of 2644 participants.

The Jackson Heart Study employs a multidimensional, multimodal data collection framework and gathers data across biological and social environmental levels. It combined standardised medical assessments, including mercury sphygmomanometers with ambulatory blood pressure monitoring for haemodynamic evaluation and enzyme colorimetry with ultracentrifugation for precise lipid profile quantification, and advanced imaging techniques (3D echocardiography for cardiac structural quantification and carotid ultrasound for atherosclerosis assessment) to collect comprehensive data spanning biological metrics (*e.g.* haemodynamics, lipid profiles, cardiac structure), behavioural factors (*e.g.* activity levels, medication adherence), and environmental variables (*e.g.* living conditions, healthcare access). To ensure data integrity, all laboratory protocols were standardised and certified by the US Centers for Disease Control and Prevention, while the imaging personnel underwent uniform certification training [18,19].

### Study population and variables

We considered all patients enrolled in the Jackson Heart Study and included those with CHF at baseline, those who participated in the first and second follow-up visits (V2 and V3), or those with electronic medical records.

The predicted outcome was the likelihood of mortality due to CHF, defined as the occurrence of death within 10 years of the follow-up period. The variables, as categorised across the five health ecological strata, encompassed a total of 58 variables (Table S1 in the **Online Supplementary Document**). These strata included personal characteristics, living conditions (*e.g.* diet, exercise, sleep, and psychological factors), interpersonal relationships, work and residential environments, and macro-level policies.

### Statistical analysis

#### Missing and standardised data handling, and delineator data set

Given the significant amount of missing data from the Jackson Heart Study, which could have biased our outcomes, we excluded data sets with more than 30% missing values based on the methods of prior studies [20,21] and subsequently employed multiple imputation using chained equations to impute any missing values [22]. We also used the standard scaler technique to standardise the diverse data from the cohort [18]. Lastly, we split the data set into 7:3 (*i.e.* 70%:30%) training and validation data sets.

#### Feature selection and collinearity check

We analysed indicators that may affect target variables using random forest (RF) and k-nearest neighbour (KNN) methods. The RF is an integrated learning algorithm that constructs multiple decision trees and combines their predictions into its final output [23]. In doing so, it employs random sampling and random selection of features, resulting in high accuracy and robustness. The RF-based feature selection process starts with bootstrapping, whereby the original training set is subjected to random put-back sampling operation and ‘n_tree’ repetitions of sampling are carried out, with ‘m’ samples extracted each time to construct ‘n_tree’ training subsets. Second, relying on these ‘n_tree’ training subsets, train them one by one, and finally obtain ‘n_tree’ corresponding decision tree models. The generated decision trees are then integrated into an RF, and when facing the classification task, multiple tree classifiers are used to construct the final classification result.

The KNN algorithm, meanwhile, belongs to the instance-based learning methods; it relies on calculating the distance between the test sample and the samples in the training set, identifying the KNNs, and then completing the classification task based on the classes to which these neighbours belong [24]. It has some limitations when dealing with high-dimensional data: for example, its high computational complexity means that the algorithm needs to consume more computational resources and time when dealing with a large amount of data. It is simultaneously susceptible to noise, which may interfere with the selection of neighbours, thus affecting the classification results. For this reason, feature selection becomes particularly critical for the KNN algorithm. Through reasonable feature selection, the dimensionality of the data can be effectively reduced, decreasing the amount of unnecessary computation, which in turn significantly improves the model’s performance and efficiency.

To mitigate the risk of overfitting, we generated a feature heat map and sequentially eliminated features with high collinearity.

#### Imbalance data handling

We used five techniques to handle the unbalanced data sets: oversampling [25], undersampling [26], adaptive synthetic sampling (ADASYN) [27], synthetic minority oversampling technique (SMOTE) [28], and synthetic minority oversampling technique and edited nearest neighbours (SMOTE-ENN) [29]. We contrasted the resulting models with those built on the original data set.

#### Model construction

We used Python, version 3.12.0 (Python Software Foundation, Wilmington, Delaware, USA) in the PyCharm IDE, version 2023.2.3 (JetBrains, Prague, Czech Republic) to construct seven ML models: decision tree (DT), RF, extreme gradient boosting (XGBoost), adaptive boosting (AdaBoost), support vector machine (SVM), naive Bayes (NB), and multilayer perceptron (MLP). We evaluated the performance of the models using the receiver operating characteristic (ROC) curve and the area under the curve (AUC) and calculated their accuracy, sensitivity, and the F1 score of the predictions. After selecting the optimal model, we performed a 10-fold cross-validation using the training and validation data sets.

## RESULT

### Patient characteristics

**Figure 1:**
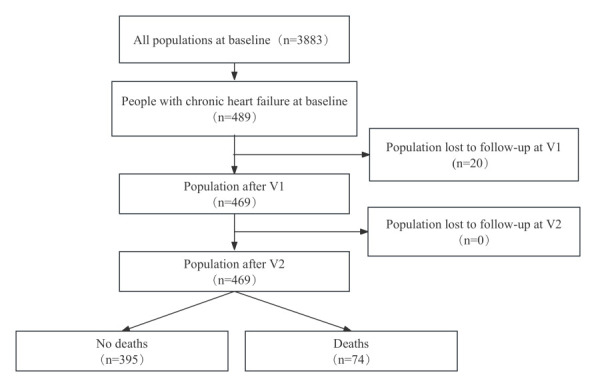
Study flowchart.

Our initial sample consisted of 489 patients with heart failure at baseline (V1), excluding 20 who were lost to V2 and V3 and did not have any associated events (death, readmission, myocardial infarction, stroke), leaving 469 participants for analysis (**Figure 1**). We randomly divided the sample into the training set (n = 328, 70%) and validation set (n = 141, 30%). The amount of missing data for vitamin D2 intake, vitamin D3 derivative intake, shortness of breath, walking 100 m with wheezing, and loneliness was greater than 30%, so we excluded them from the analysis (Figure S1 in the **Online Supplementary Document**). The mean age of the study population was 54.08 years (standard deviation = 11.63), with 328 (69.9%) females and 141 (30.1%) males.

### Univariate analysis of the baseline characteristics

The difference in 22 factors was statistically significant (*P* < 0.05) when comparing patients with and without recorded deaths in CHF, including smoking (*P* = 0.006), glucose-lowering medication use (*P* < 0.001), antiarrhythmic medication use (*P* < 0.001), history of vertigo (*P* < 0.001), history of cardiovascular surgery (*P* < 0.001), history of coronary artery disease (*P* < 0.001), income (*P* = 0.043), occupation (*P* = 0.016), educational qualifications (*P* < 0.001), foot/ankle oedema (*P* = 0.009), marital status (*P* = 0.016), paradoxical movement of the left ventricular wall (*P* < 0.001), age (*P* < 0.001), systolic blood pressure (*P* < 0.001), glycosylated haemoglobin (*P* < 0.001), triglycerides (*P* = 0.032), left ventricular ejection fraction (*P* = 0.043), left ventricular diastolic diameter (*P* < 0.001), left ventricular mass (*P* < 0.001), favourite food shop in three kilometre radius (*P* = 0.019), percentage of population living in poverty in the region (*P* = 0.001), and ratio of peak ‘e’ to peak ‘a’ of antegrade mitral flow (*P* = 0.029) (Tables S2 and S3 in the **Online Supplementary Document**).

### Characteristic selection of factors influencing mortality in patients with CHF

We used RF and KNN for feature selection, with the models trained using root mean square error (RMSE) [30].

We included 24 feature after RF selection: left ventricular mass, BMI, systolic blood pressure, glomerular filtration rate, favourite food stores within three kilometres, proportion of the population living in poverty in the area, dark-coloured green vegetables, E/A ratio, left ventricular diastolic diameter, glycated haemoglobin, peak early diastolic velocity of mitral annulus, high-density lipoprotein, ejection fraction, age, low-density lipoprotein, triglyceride, history of cardiovascular surgery, egg, total cholesterol, hours of actual sleep at night, vitamin D3, ultrasensitive c-reactive protein, heart rate, and left ventricular regional wall motion (Figure S2 in the **Online Supplementary Document**).

We included 18 features after KNN selection: age, smoking, antihypertensive medication, hypoglycaemic medication, antiarrhythmic medication, systolic blood pressure, glycated haemoglobin, glomerular filtration rate, left ventricular ejection fraction, left ventricular diastolic diameter, left ventricular mass, history of vertigo, history of cardiovascular surgery, history of coronary artery disease, occupation, educational level, percentage of people in poverty in the area, percentage of people in poverty in the area, and paradoxical movement of the wall of the left ventricle (Figure S3 in the **Online Supplementary Document**).

**Figure 2:**
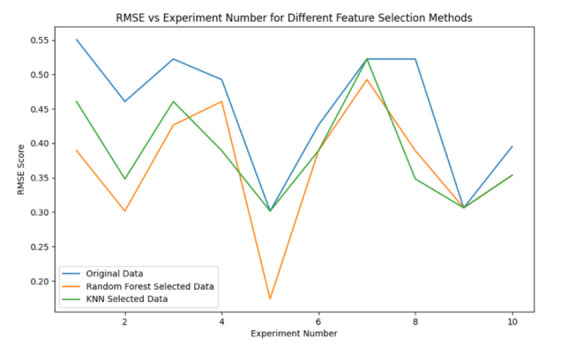
RMSE results of the original data and different feather selection.

We used 10-fold cross-validation to compare the feature selection methods, and we calculated and plotted the average RMSE (**Figure 2**; Table S4 in the **Online Supplementary Document**). The results after RF feature selection were better than those obtained after KNN feature selection. The average RMSE after 10-fold cross-validation for RF feature selection is 0.368.

Based on the characteristic heat map (Figure S4 in the **Online Supplementary Document**), we note that total cholesterol and low-density lipoprotein have high collinearity. A new data set was formed by deleting the feature total cholesterol with low feature importance (*i.e.* with feature importance of 0.024819) (**Table 1**; Figure S5 in the **Online Supplementary Document**).

**Table 1:** Factor hierarchical summarisation table

Health ecology	Factors
**Individual level**	Age, body mass index, use of antihypertensive, hypoglycaemic, and antiarrhythmic medications; systolic blood pressure; glycated haemoglobin; glomerular filtration rate; left ventricular ejection fraction; left ventricular diastolic diameter; left ventricular mass; high-density lipoproteins; low-density lipoproteins; triglycerides; history of cardiovascular surgery; and parameters of mitral annular early diastolic velocity, paradoxical left ventricular wall motion, and the ratio of peak e to peak a of antegrade mitral flow
**Community level**	Proportion of individuals living in poverty within the residential area
**System or service level**	Difficulty in obtaining the three-kilometre distance from the patient's preferred food outlet

### Oversampling processing

We evaluated all data balancing techniques using the accuracy and AUC of the seven models, and selected optimal data balancing technique for subsequent model construction (**Table 2**). When analysing by combining the accuracy and AUC metrics, it is found that in the RF, the accuracy of SMOTE-ENN reaches 78.95%, which is much higher than the 63.64% of undersampling. Meanwhile, the AUC reaches 0.85, indicating that the model has a strong ability to distinguish between sample classes under this combination and can better match RF to improve the classification accuracy. After combining the results, SMOTE-ENN was superior to the other data balancing techniques used in this study.

**Table 2:** Comparison of imbalanced data handling techniques across each machine learning algorithm

Algorithms	Performance metrics	Unbalanced data	Under-sampling	Over-sampling	ADASYN	SMOTE	SMOTE-ENN
DT	Accuracy	71.83%	77.27%	58.33%	56.78%	59.17%	64.47%
	AUC	0.50	0.77	0.58	0.56	0.59	0.62
RF	Accuracy	84.51%	63.64%	50.00%	58.47%	63.33%	78.95%
	AUC	0.55	0.73	0.69	0.69	0.76	0.85
XGBoost	Accuracy	81.69%	63.64%	45.83%	68.64%	70.00%	81.58%
	AUC	0.62	0.64	0.62	0.71	0.75	0.83
AdaBoost	Accuracy	78.87%	63.64%	47.50%	55.08%	55.83%	60.63%
	AUC	0.48	0.54	0.45	0.59	0.62	0.65
SVM	Accuracy	84.51%	63.64%	59.17%	59.32%	61.67%	65.79%
	AUC	0.42	0.39	0.65	0.63	0.65	0.78
NBM	Accuracy	78.65%	59.09%	59.17%	59.32%	62.50%	71.05%
	AUC	0.66	0.63	0.68	0.77	0.81	0.83
MLP	Accuracy	67.61%	50.00%	65.00%	62.71%	65.00%	63.16%
	AUC	0.64	0.51	0.68	0.67	0.68	0.65

AdaBoost – adaptive boosting, ADASYN – adaptive synthetic sampling, AUC – area under curve, DT – decision tree, MLP – multilayer perceptron, NBM – naive Bayesian model, RF – random forest, SMOTE – synthetic minority oversampling technique, SMOTE-ENN – SMOTE combined with edited nearest neighbors, SVM –support vector machine, XGBoost –eXtreme gradient boosting

### Model construction results

**Figure 3:**
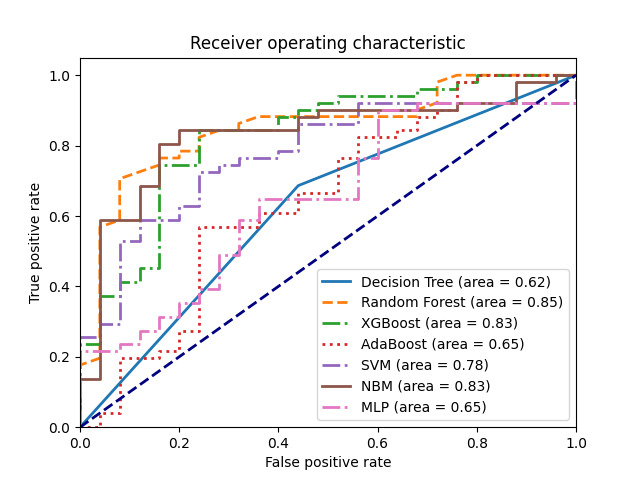
Comparison of ROC curves of different models.

Among all the models, the XGBoost model was the most effective, with an AUC value of 0.83, an accuracy of 81.58%, a precision of 0.87, a recall of 0.84, and an F1 value of 0.86 (**Figure 3**, **Table 3**).

**Table 3:** Performance comparison of different models

Algorithms	AUC	Accuracy	Precision	Sensitivity	F1
DT	0.62	64.47%	0.76	0.69	0.72
RF	0.85	78.95%	0.82	0.88	0.85
XGBoost	0.83	81.58%	0.87	0.84	0.86
AdaBoost	0.65	60.63%	0.74	0.63	0.68
SVM	0.78	65.79%	0.68	0.92	0.78
NBM	0.83	71.05%	0.73	0.90	0.81
MLP	0.65	63.16%	0.73	0.73	0.73

AdaBoost – adaptive boosting, AUC – area under the curve, DT – decision tree, MLP – multilayer perceptron, NBM – naive Bayesian model, RF – random forest, SVM – support vector machine, XGBoost – eXtreme gradient boosting

The results of the 10-fold cross-validation show that the AUC value is 0.99, the accuracy is 96.84%, the precision is 0.98, the recall is 0.97, the F1 value is 0.98, and the ROC curve is shown (Figure S6 in the **Online Supplementary Document**).

To enhance the performance of the model, we employed a grid search methodology combined with 10-fold cross-validation to fine-tune the parameters of the XGBoost model. A defined search space is established for key parameters, including ‘n_estimators’, ‘learning_rate’, ‘max_depth’, and ‘subsample’, with the AUC and accuracy serving as the evaluation metrics for assessing the various parameter combinations. Following the optimisation process, the model’s accuracy improved to 87.32%.

## DISCUSSION

### Risk factor analysis for the mortality of CHF based on the health ecology

Here we employed two methodologically different feature selection algorithms and compared them to delineate 23 risk factors predictive of mortality for patients with CHF. Drawing from the health ecology theory, we implemented a systematic and hierarchical categorisation of the identified risk factors to elucidate their mechanisms and interconnections across various levels. This was done to develop a more personalised and targeted mortality risk assessment protocol for CHF patients, in order to inform control and early-stage interventions with adjusted treatment strategies.

We incorporated 19 variables with respect to individual risk factors. Research has demonstrated that health levels decline with age and are prone to loss of compensation due to multiple factors such as age-related depletion of physiological reserves, comorbidity with other conditions, and precipitating factors like infection/fatigue, leading to increased rates of heart failure and unfavourable outcomes [31,32]. Antihypertensive medication and systolic blood pressure serve as indicators of blood pressure status; high-density lipoproteins, low-density lipoproteins, and triglycerides reflect lipid status; antihypertensive and glycated haemoglobin medications correspond to blood glucose control; and the glomerular filtration rate measures renal function. Studies have shown that an increased burden of chronic diseases (*e.g.* diabetes mellitus, hypertension, hyperlipidaemia, renal disease) correlates with a higher mortality risk among patients with CHF [33]. Antiarrhythmic medications, left ventricular ejection fraction, left ventricular diastolic diameter, left ventricular mass, peak mitral annular early diastolic velocity of motion, left ventricular wall motion, and the ratio of peak ‘e’ to peak ‘a’ of antegrade mitral flow are indicative of cardiac structural changes. Previous research has highlighted the parallel between ventricular remodelling and the pathophysiological progression caused by ventricular decompensation, including increased afterload and right ventricular hypertrophy, which are independent predictors of mortality in CHF [34,35].

With respect to individual behavioural characteristics, three risk factors were considered: intake of dark green plants, consumption of eggs, and night sleep duration. Behavioural characteristics have a significant influence on health outcomes. For elderly individuals, adhering to a healthy lifestyle is linked to a diminished risk of mortality [36]. The consumption of dark green plants and eggs is indicative of nutritional status, as it has been established that vegetables are a rich source of dietary fibre, catechins, and theaflavins, which exert significant anti-inflammatory and antioxidant properties, contributing to the maintenance of homeostasis and the overall health of bodily functions [37]. Consequently, increased intake of dark green vegetables may reduce the risk of mortality from CHF. Previous research has demonstrated a U-shaped relationship between egg consumption and the risk of cardiovascular disease (including coronary heart disease and stroke) and total mortality, suggesting that excessive or insufficient egg intake can be detrimental to cardiovascular health [38]. Moderate egg consumption has been shown to effectively reduce the risk of mortality from CHF [39]. Sleep has also been identified as an independent risk factor for CHF [40]. The findings of the most recent prospective cohort study further indicate a U-shaped relationship between sleep duration and cardiovascular disease risk [41]. As such, it is recommended that individuals with CHF limit their sleep duration to between 6.5 and 8 hours [42].

We considered two risk factors in the context of living environment and working conditions: the three-kilometre distance from the patients’ preferred food outlet and the proportion of individuals living in poverty within the residential area. The distance to a three-kilometre preferred food outlet serves as an indicator of the patient's dietary status, which is congruent with the dietary behaviour within the stratum of individual behavioural characteristics. The proportion of individuals living in poverty within the residential area reflects the patients’ local economic status. Socioeconomic status is typically assessed through factors such as income, education, or occupation [43]. Moreover, favourable living and working conditions correlate with enhanced disease coping abilities, encompassing access to quality health care, comprehensive health insurance, regular health screenings, and availability of essential rehabilitation services and long-term care [44]. There is a well-documented association between low socioeconomic status and an increased risk of mortality from cardiovascular disease, particularly CHF [45]. Individuals with low socioeconomic status may face barriers in accessing needed medical interventions for care [46].

The model in our study has not accounted for factors at the interpersonal relationship level, a possibility originating from the constraints of the data collection dimensions. Prior research utilised basic variables, such as marital status and loneliness, to measure interpersonal social networks but falls short of elaborating on critical dimensions, including the intensity of emotional support and the frequency of community interactions [47]. Consequently, this level of information is obscured within the high-dimensional feature space by physiological indicators [48]. Moreover, CHF, as an end-stage organic cardiovascular disease, exhibits direct physiological effects, such as myocardial remodelling and haemodynamic disturbances, that surpass the indirect influence of psychosocial factors [49]. This discrepancy may diminish the predictive ability of interpersonal relationship variables [50]. The absence of macro-level policy factors may be attributed to the protracted nature of policy impacts, which typically exhibit a lag of several years to decades, making them challenging to discern within our study’s 10-year follow-up period. Moreover, the quantification dilemma associated with policy variables, such as the lack of standardised assessment indicators for policy implementation effects, may result in the exclusion of this information during feature selection [51,52]. These findings indicate that future research should enhance the precision of social ecological data collection and integrate longer follow-up periods with policy effect quantification techniques, thereby fully determining the relationships and interactions between multi-dimensional risk factors.

### Prediction model construction for the occurrence of CHF based on ML

While traditional regression methods have been predominantly utilised in clinical practice for identifying risk factors associated with CHF mortality [53]. Nevertheless, there remains a dearth of standardised, precise predictive models designed for this purpose. Although traditional regression methods yield predictive results with varying degrees of reference value, they struggle to minimise the discrepancy between predicted and actual values.

Here we used the SMOTE-ENN approach to address issues with our unbalanced data sets. Through the generation of high-quality synthetic examples and the enhancement of nearest-neighbour samples, SMOTE-ENN can effectively enhance the classification performance of the model on underrepresented classes, particularly within unbalanced data sets [54]. Research suggests that balanced data sets are crucial for constructing predictive models related to disease occurrence and progression, thereby improving model accuracy and performance [55].

We found the XGBoost model to be the most effective, with an AUC value of 0.83, an accuracy of 81.58%, a precision of 0.87, a recall of 0.84, and an F1 value of 0.86. An AUC value between 0.9 and 1 is indicative of highly accurate prediction [56]. Our findings contrast some, but agree with other research. One prior study constructed a multimodal deep learning model to predict CHF mortality risk; their model achieved an AUC of 0.838, which is lower than our best model [57]. Yang and colleagues [58] used the Cox model to predict the  CHF mortality risk within 30 days or 1 year; they arrived at two models with AUCs of 0.778 and 0.738, respectively. This would indicate that ML is better than the traditional method of using Cox models to predict the mortality risk of CHF patients.

We note, however, that the implementation of the SMOTE-ENN technique is associated with the risk of bias regarding the authenticity of synthetic samples, as well as the possibility of omitting critical samples located in boundary regions [59,60]. Similarly, the XGBoost model is susceptible to overfitting when dealing with high-dimensional features, and it sees challenges related to the balance between computational resource requirements and practical clinical application [61,62]. These factors pose significant obstacles to the effective translation of models developed through research into clinical practice.

### Limitations and suggestions for future steps

We note several limitations to this study. We used the Jackson Heart Study database as our primary data source. which encompasses a decade-long cohort. There is currently is no updated data set available, preventing external validation using the same cohort and constraining generalisability of the findings. Nevertheless, an investigation conducted by our research team has demonstrated promising external validation results (data not yet published). Furthermore, the database exclusively includes African American adults, does not represent low-income regions or countries, and does not reflect the diversity of CHF patients globally, diminishing the broader applicability of our predictive model. Furthermore, while we incorporated variables from the five dimensions of health ecology, our analysis did not consider factors at the ‘interpersonal’ and ‘social environment’ levels, thereby rendering the theoretical framework incomplete. Additionally, the data set does not contain global socioeconomic and environmental variables, such as infectious comorbidities, malnutrition, and environmental exposures, which precluded an examination of the potential influence of these factors on CHF mortality.

Future research should prioritise including populations from other regions regions and other economic and cultural contexts. It is also important to develop a modified version of SMOTE-ENN that integrates insights from heart failure research. Additionally, investigating lightweight ensemble models designed to reduce computational costs could help address the limitations of the previously mentioned algorithms. Lastly, external validation of the predictive models should be conducted using retained cohorts or independent data sets to enhance their clinical relevance.

## CONCLUSIONS

We used ML techniques to identify risk factors associated with mortality in patients with CHF, which we systematically categorised based on the health ecology theory. We identified 24 traits encompassing three levels. At the individual physiological and clinical level, these factors include age, systolic blood pressure, glycosylated hemoglobin, glomerular filtration rate, left ventricular ejection fraction, left ventricular end-diastolic diameter, left ventricular mass, left ventricular wall motion abnormalities, a history of dizziness, a history of cardiovascular surgery, and a history of coronary heart disease. These are closely associated with the burden of chronic diseases and structural cardiac alterations, influencing disease progression and outcomes. At the individual behavioral and treatment level, we noted factors such as smoking status and use of antihypertensive, hypoglycemic, and antiarrhythmic medications reflect lifestyle habits and therapeutic management that impact health status. At the social and environmental level, we found factors such as occupation, educational background, and the proportion of the regional poor population are linked to socioeconomic status and regional development, with lower socioeconomic status being strongly associated with an elevated risk of mortality due to CHF.

We constructed a novel dataset based on these screening factors. After evaluating several data balancing techniques on this new dataset, SMOTE-ENN was determined to be the most effective. We then developed multiple models using SMOTE-ENN-processed data, with the XGBoost model exhibiting the highest efficacy with an AUC of 0.87, an accuracy of 88.64%, and superior performance across all metrics. Future research should focus on further exploring and optimising these methodologies to enhance the prediction of mortality risk in CHF and their application in clinical practice.

## Additional material


Online Supplementary Document
