# Supplementary material for: Machine learning-based risk factor analysis and prediction model construction for mortality in chronic heart failure
Source: J Glob Health. 2025 Sep 12;15:04242. doi: 10.7189/jogh.15.04242 (PMC12427600; doi:10.7189/jogh.15.04242)
Supplement: Online Supplementary Document [file jogh-15-04242-s001.pdf]

Supplement to: Xu Q, Yu R, Cai X, Chen G, Zheng Y, Xu C, Sun J. Machine learning-based risk factor analysis and prediction model construction for mortality in chronic heart failure. J Glob Health. 2025;15:04242.

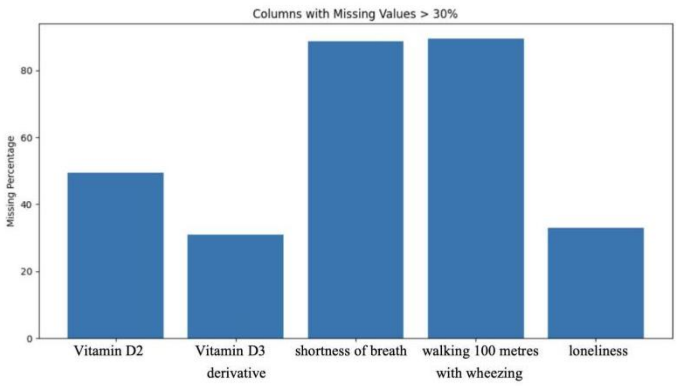

Figure S1 Data visualizations with greater than 30% missing graphs

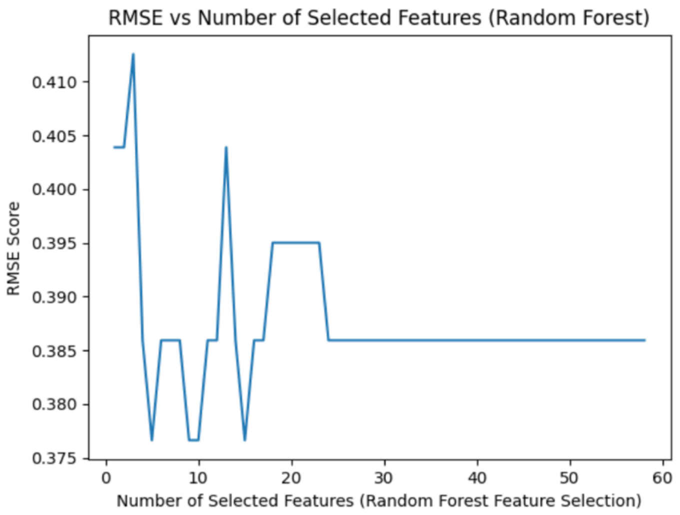

Figure S2 RMSE result of RF

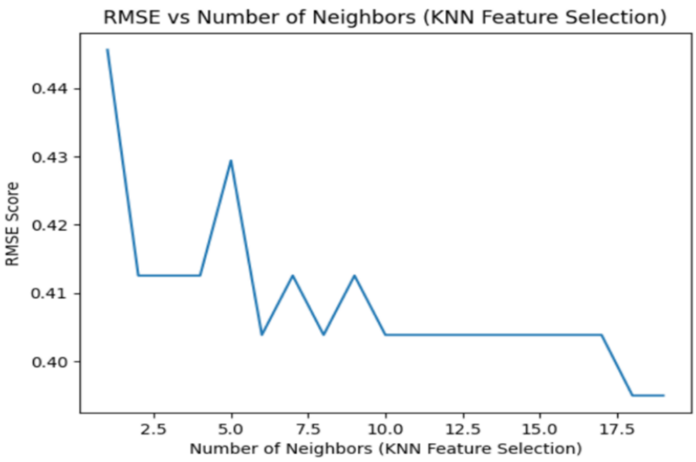

Figure S3 RMSE result of KNN

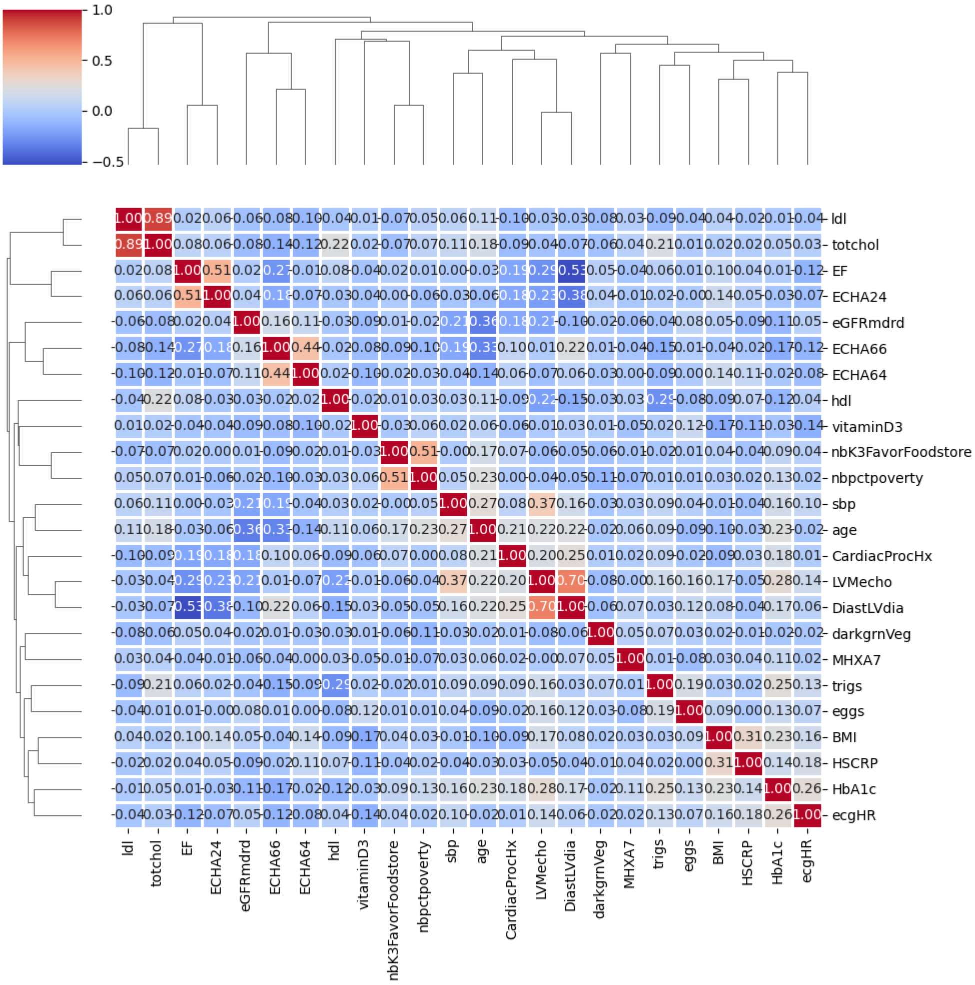

Figure S4 The characteristic heat map

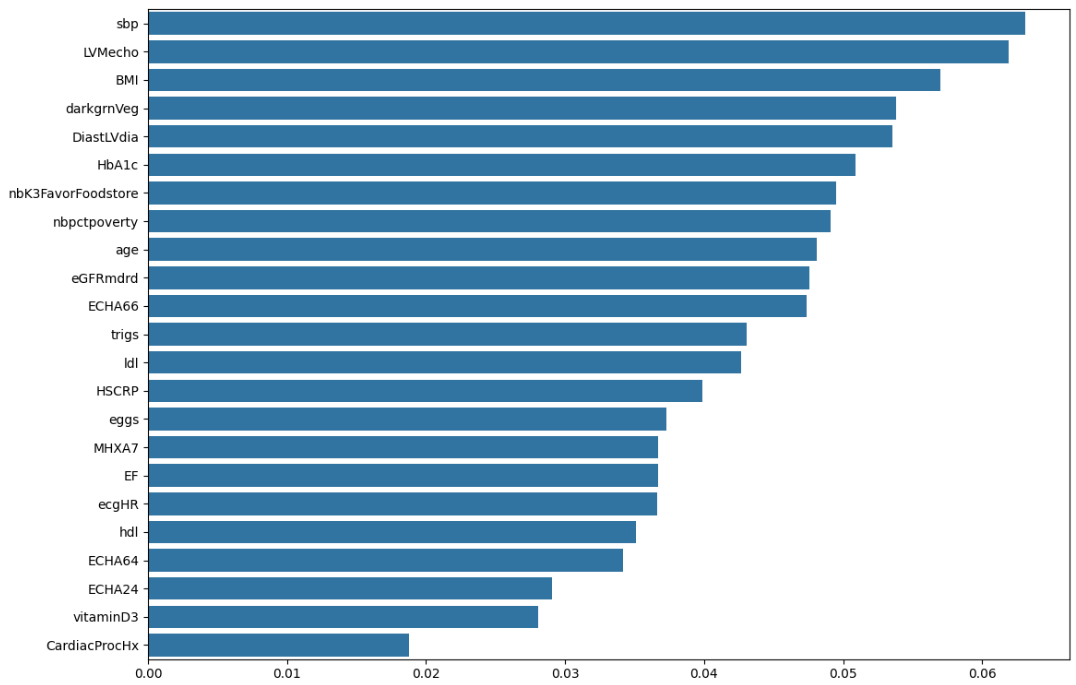

Figure S5 The importance of feathers

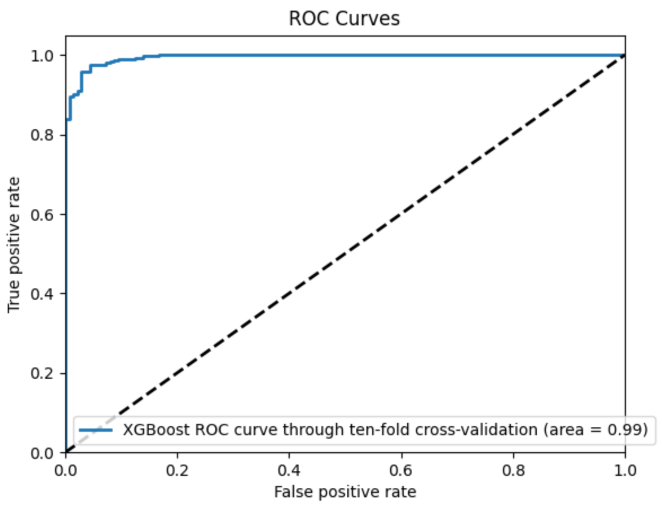

Figure S6 ROC curves of Adaboost on the whole data through ten-fold cross-validation

Table S1 Predictor Variables

Name	Label	Unit
	Personal traits	
age	age	year
gender	male	/
Body Mass Index(BMI)	BMI	kg/m^2
Systolic Blood Pressure	sbp	mmHg
Glycosylated Hemoglobin	HbA1c	%
Low Density Lipoprotein (LDL)	ldl	mg/dL

High Density Lipoprotein (HDL)	hdl	mg/dL
Fasting Triglyceride Level	trigs	mg/dL
Fasting Total Cholesterol	totchol	mg/dL
High Sensitivity C-Reactive Protein	HSCRP	Serum mg/dL
Glomerular Filtration Rate	eGFR	ml/min/1.73 m2
Ejection Fraction	EF	/
Left Ventricular Diastolic Diameter	DiastLVdia	mm
Left Ventricular Mass	echoLV	g
Heart Rate	ecgHR	bpm
Self-reported dialysis	DialysisEver	/
Chronic Kidney Disease History	CKDHx	/
History of Stroke	strokeHx	/
Coronary Heart Disease Status/History	CHDHx	/
Self-Reported History of MI	MIHx	/
Self-Reported history of Cardiac Procedures	CardiacProcHx	/
Left Ventricular Wall Anomalous Motion	ECHA24	/
Peak early diastolic velocity of mitral annulus	ECHA64	m/sec
The Ratio Of Early Maximal Ventricular Filling Velocity To Atrial Maximal Ventricular Filling Velocity	ECHA66	/
Ever awakened by trouble breathing?	MHXA48	/
Ever had swelling of feet or ankles?	MHXA49	/
Told that you stop breathing in your sleep.	MHXA2	/
Chest ever sound wheezy without cold?	RPAA8	/
Troubled by shortness of breath?	RPAA19	/
Stop for breath after walking 100yds?	RPAA22	/
Hypertensive Medicine	BPmeds	/
Diabetes Medicine	DMmeds	/
Statin Medicine	statinMeds	/
Estrogen or progesterone	hrtMeds	/
Beta-Blocker (Medicine)	betaBlkMeds	/
Calcium Channel Blockers	calBlkMeds	/
Diuretic Medicine	diureticMeds	/
Antiarrhythmic Medicine	antiArythMeds	/

Personal living conditions

Alcohol drinking in the past 12 months	alc	/
25(OH) Vitamin D2	VitaminD2	ng/mL
25(OH) Vitamin D3	VitaminD3	ng/mL
ep-25(OH) Vitamin D3	VitaminD3epimer	ng/mL
Dark-green Vegetables	darkgrnVeg	/
Eggs	eggs	/
Fish	fish	/
Hours of actual sleep at night?	MHXA7	hours
Rate your sleep quality overall	MHXA6	/
Self-Reported History of Cigarette Smoking	eversomker	/
Stress	perceivedStress	/
minutes walk or run	PACA1	/
	Interpersonal relationships	
Married/Single/Divorced/Widowed	SOCA1A	/
Depression	depression	/
Lonely	CESA14	/
Threatened/harassed	Threatened/harassed	/
Stress living in neighborhood	STSA3	
	work and living conditions	
Place usually go(medical institution)	HCAA2A	/
Difficulty in obtaining health service	HCAA6	/
Satisfied with doctor	HCAA11	/
Income	Income	/
Occupation	occupation	/
Education	edu3cat	/
	Policy	
Health Insurance	Insured	/

Table S2 One-way analysis of the baseline characteristics

Variables	Death		Non-Death		$\chi2$	*P*
	n	%	n	%		
Gender					3.480	0.062
Woman	45	9.59%	112	23.88%		
Man	29	5.18%	283	60.34%		
Alcohol					1.716	0.190
Yes	29	6.18%	187	39.87%		
No	45	9.59%	207	44.14%		
Smoking					7.558	0.006
Yes	38	8.10%	136	29.00%		
No	36	7.68%	258	55.01%		

Hearth Insurance					6.563	0.10
Yes	57	12.15%	245	52.24%		
No	16	3.41%	147	31.34%		
Diabetes Medicine					19.344	<0.001
Yes	29	6.18%	58	12.37%		
No	43	9.17%	324	69.08%		
Statin Medicine					1.129	0.288
Yes	14	2.99%	57	12.15%		
No	58	12.37%	335	71.43%		
Estrogen or progesterone					1.374	0.241
Yes	10	2.13%	74	15.78%		
No	62	13.22%	300	63.97%		
Beta-Blocker (Medicine)					1.999	0.157
Yes	15	3.20%	56	11.94%		
No	53	11.30%	313	66.74%		
Calcium Channel Blockers					2.261	0.133
Yes	23	4.90%	92	19.62%		
No	45	9.59%	275	58.64%		
Diuretic Medicine					2.315	0.128
Yes	37	7.89%	163	34.75%		
No	32	6.82%	210	44.78%		
Antiarrhythmic Medicine					12.066	<0.001
Yes	15	3.20%	30	6.40%		
No	57	12.15%	362	77.19%		
History Of Dialysis					2.951	0.086
Yes	3	0.64%	4	0.85%		
No	71	15.14%	391	83.37%		
History Of Kidney Disease					0.077	0.782
Yes	8	1.71%	39	8.32%		
No	65	13.86%	355	75.69%		
History Of Stroke					0.561	0.454
Yes	6	1.28%	23	4.90%		
No	68	14.50%	372	79.32%		
History Of Vertigo					15.265	<0.001
Yes	20	4.26%	41	8.74%		
No	54	11.51%	354	75.48%		
History Of Cardiovascular Surgery					22.685	<0.001
Yes	24	5.18%	44	9.38%		
No	50	10.67%	350	74.63%		
History Of Coronary Heart Disease					16.442	<0.001
Yes	22	4.70%	46	9.81%		
No	52	11.09%	349	74.41%		
Hearth Insurance					2.073	0.150
Yes	58	12.37%	59	12.58%		

No	16	3.41%	336	71.64%		
Threats/harassment					3.566	0.735
Several times a day	2	0.43%	4	0.85%		
Almost every day	2	0.43%	8	1.71%		
At least once a week	1	0.21%	16	3.41%		
A few times a month	2	0.43%	13	2.77%		
A few times a year	6	1.28%	23	4.90%		
Less than a few times a year	9	1.92%	55	11.73%		
Never	52	11.09%	267	57.56%		
Income					8.143	0.043
Poor	19	4.05%	66	14.07%		
Lower-middle	16	3.41%	97	20.68%		
Upper-middle	19	4.05%	92	19.62%		
Affluent	6	1.28%	9	1.92%		
Occupation					5.778	0.016
Management/Professional	18	3.84%	125	26.65%		
Service	22	4.69%	119	25.37%		
Sales	7	1.49%	63	13.43%		
Farming	0	0.00%	1	0.21%		
Construction	7	1.49%	27	5.76%		
Production	20	4.26%	58	12.38%		
Military	0	0.00%	0	0.00%		
Sick	0	0.00%	0	0.00%		
Unemployed	0	0.00%	1	0.21%		
Retired	0	0.00%	0	0.00%		
Student	0	0.00%	0	0.00%		
Education					17.032	<0.001
Less than high school	27	5.76%	66	14.07%		
High school graduate/GED	16	3.41%	86	18.34%		
Attended vocational school, trade school, or college	30	6.40%	242	51.60%		
Stress living in neighborhood					6.545	0.088
Not Stressful	43	9.17%	273	58.21%		
Mildly Stressful	13	2.77%	67	14.29%		
Moderately Stressful	10	2.13%	25	5.33%		
Very Stressful	8	1.71%	28	59.70%		
Minutes of walking or running					3.691	0.449
Less than 5 minutes	38	8.10%	176	37.53%		
At least 5 but less than 15 minutes	8	1.71%	74	15.78%		
At least 15 but less than 30 minutes	12	2.56%	56	11.94%		
At least 30 but less than 45	8	1.71%	35	7.46%		

minutes						
At least 45 minutes	8	1.71%	54	11.51%		
Place usually go					0.257	0.612
Walk-in clinic	10	2.13%	37	7.89%		
Health Maintenance Organization clinic	0	0.00%	1	0.21%		
Hospital clinic	13	2.77%	44	9.38%		
Neighbourhood health center	3	0.64%	45	9.59%		
Hospital emergency room	1	0.21%	1	0.21%		
Public health department clinic	0	0.00%	3	0.64%		
Company or industry clinic	1	0.21%	7	1.49%		
Doctor's office	42	8.96%	206	43.92%		
Other	0	0.00%	3	0.64%		
Difficulty in obtaining health service					1.092	0.296
Very hard	10	2.13%	45	9.59%		
Fairly hard	7	1.49%	35	7.46%		
Not too hard	17	3.62%	63	1.24%		
Not hard at all	40	8.53%	248	52.88%		
Satisfied with doctor					1.210	0.086
Very satisfied	47	10.02%	232	49.47%		
Somewhat satisfied	22	4.69%	129	27.50%		
Somewhat dissatisfied	2	0.43%	12	2.56%		
Very dissatisfied	2	0.43%	5	1.07%		
Not sure	1	0.21%	5	1.07%		
Ever awakened by trouble breathing					3.065	0.080
Yes	71	15.14%	392	83.58%		
No	3	0.64%	3	0.64%		
Rate your sleep quality overall					8.570	0.073
Excellent	2	0.43%	20	4.26%		
Fair	30	6.40%	122	26.01%		
Good	15	3.20%	123	2.62%		
Poor	19	4.05%	68	14.50%		
Very good	8	1.71%	62	13.22%		
Ever had swelling of feet or ankles					6.893	0.009
Yes	60	12.79%	259	55.22%		
No	14	2.99%	136	29.00%		
Chest ever sound wheezy without cold					11.00	0.294
Yes	18	3.84%	120	25.59%		
No	56	11.94%	275	58.64%		
Marriage					12.184	0.016
Divorced	19	4.05%	65	13.86%		
Married	26	5.54%	201	42.86%		

Unmarried	7	1.49%	57	12.15%		
Separate	4	0.85%	20	4.26%		
Widowed	18	3.84%	51	10.87%		
LV regional wall motion					24.265	<0.001
Abnormal	8	1.71%	7	1.49%		
Border	4	0.85%	5	1.49%		
Normal	57	12.15%	374	79.74%		
Can't Assess	1	0.21%	4	0.85%		

Table S3 One-way analysis of the baseline characteristics

Variables	Death $\bar{x} \pm s$/[$M(P_{25}, P_{75})$]	Non-Death $\bar{x} \pm s$/[$M(P_{25}, P_{75})$]	Z Value	P
Age（Year）	60.50 (53.00, 67.25)	53.00 (44.00, 62.00)	-4.735	<0.001
BMI（kg/m^2）	33.77 (28.14, 39.07)	33.07 (28.89, 37.08)	-0.372	0.710
Systolic Blood Pressure（mmgH）	132.54 (122.45, 149.04)	122.00 (113.74, 130.25)	-4.530	<0.001
Glycosylated Hemoglobin（%）	6.00 (5.63, 7.28)	5.60 (5.30, 6.00)	-3.841	<0.001
Low Density Lipoprotein（mg/dL）	107.00 (97.00, 137.00)	118.00 (98.00, 143.00)	-1.658	0.097
High Density Lipoprotein（mg/dL）	46.50 (39.50, 51.75)	50.00 (40.00, 61.00)	-1.481	0.139
Fasting Triglycerides（mg/dL）	98.00 (74.50, 148.75)	91.00 (65.00, 122.00)	-2.140	0.032
Fasting Total Cholesterol（mg/dL）	180.00 (160.25, 214.75)	192.00 (166.00, 219.00)	-1.704	0.088
Ultrasensitive C-Reactive Protein（mg/dL）	0.51 (0.18, 0.91)	0.35 (0.13. 0.81)	-1.036	0.300
Glomerular Filtration Rate（mL/min）	81.71 (65.86, 98.93)	85.57 (74.49, 96.30)	-2.016	0.044
Ejection Fraction（%）	60.00 (46.25, 65.00)	65.00 (55.00, 65.00)	-2.022	0.043
Left Ventricular Diastolic Diameter	53.80 (50.18, 56.85)	50.00 (47.30, 52.20)	-4.197	<0.001
Left Ventricular Mass	185.78 (158.29, 232.54)	140.71 (124.63, 169.60)	-4.614	<0.001
Heart Rate	63.00 (59.00, 71.75)	63.00 (57.00,71.00)	-1.852	0.064
Vitamin D3（ng/mL	12.20 (9.10, 16.80)	12.00 (8.43, 16.38)	-0.211	0.833
Dark-colored Green Vegetables（ng/mL）	0.25 (0.10, 0.43)	0.24 (0.16,0.40)	-0.875	0.382
Status of Favorite Food Stores Within 3 Kilometers	0.39 (0.10, 0.58)	0.24 (0.62, 0.46)	-2.341	0.019
Status of Sports Facilities Within 3 Kilometers	0.39 (0.17, 0.76)	0.35 (0.17, 0.71)	-0.656	0.512
Stress	6.00 (3.00, 8.00)	6.00 (3.00, 11.00)	-1.019	0.308
Egg（ng/mL）	0.31 (0.06, 1.01)	0.32 (0.84, 0.63)	-0.951	0.342
Proportion of the Population Living in Poverty in the Area（%）	0.26 (0.18, 0.38)	0.22 (0.12, 0.32)	-3.224	0.001
Peak early diastolic velocity of mitral annulus（cm/s）	0.83 (0.70, 0.94)	0.87 (0.74, 0.94)	-1.293	0.196
the ratio of early maximal ventricular filling velocity to atrial maximal ventricular filling velocity (E/A)	0.98 (0.82, 1.21)	1.12 (0.94, 1.35)	-2.178	0.029
Hours of actual sleep at night (Hours)	6.00 (5.00, 7.00)	6.00 (5.00, 7.00)	-0.823	0.411

Table S4 The average RMSE of the original data and different feather selection

Method	The average RMSE
The original data	0.450
Feather selected by RF	0.368
Feather selected by PCA	0.388
